# Tetra­aqua­bis[μ-*N*-(5-nitro-2-oxido­benzyl­idene)glycylglycinato]manganese(II)dinickel(II) tetra­hydrate

**DOI:** 10.1107/S1600536810011293

**Published:** 2010-03-31

**Authors:** Yang Zou

**Affiliations:** a Chemistry Department, Zhejiang Sci-Tech University, Hangzhou 310018, People’s Republic of China

## Abstract

The two unique Ni^II^ atoms of the title complex, [MnNi_2_(C_11_H_8_N_3_O_6_)_2_(H_2_O)_4_]·4H_2_O, have a slightly distorted square-planar coordination environment with a tetra­dentate *N*-(5-nitro-2-oxidobenzyl­idene)glycylglycinate Schiff base trianion. The Ni^II^ atoms are coordinated by one phenolate O atom, one imine N atom, one amido N atom and one carboxyl­ate O atom. The Mn^II ^atom is connected *via* the carboxyl­ate groups, forming a hetero-trinuclear Ni^II^–Mn^II^–Ni^II^ system. The Mn^II^ atom is six-coordinated in an octa­hedral geometry by four O atoms from two carboxyl­ate groups and four water mol­ecules. The Ni^II^–Mn^II^–Ni^II^ hetero-trinuclear mol­ecules are stacked in the crystal and cross-linked through O—H⋯O hydrogen bonds.

## Related literature

Transition metal complexes of salicylaldehyde-peptides and salicylaldehyde-amino acid Schiff bases are non-enzymatic models for pyridoxal-amino acid systems, which are of considerable importance as key inter­mediates in many metabolic reactions of amino acids catalysed by enzymes, see: Bkouche-Waksman *et al.* (1988 ▶); Wetmore *et al.* (2001 ▶); Zabinski & Toney (2001 ▶). For the preparation, structural characterization, appropriate spectroscopy and magnetic studies of Schiff-base complexes derived from salicylaldehyde and amino acids, see: Ganguly *et al.* (2008 ▶) and references cited therein. For Schiff bases derived from simple peptides, see: Zou *et al.* (2003 ▶).
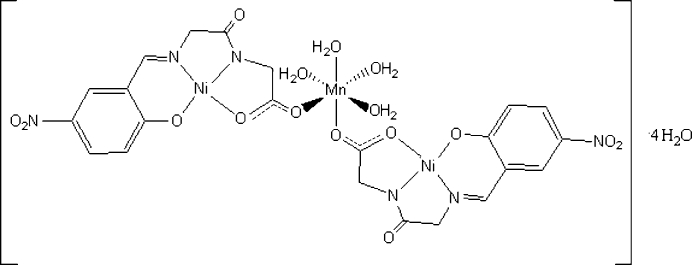


## Experimental

### 

#### Crystal data

[MnNi_2_(C_11_H_8_N_3_O_6_)_2_(H_2_O)_4_]·4H_2_O
*M* *_r_* = 872.90
Monoclinic, 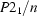

*a* = 7.250 (1) Å
*b* = 11.581 (2) Å
*c* = 38.058 (6) Å
β = 90.29 (1)°
*V* = 3195.4 (9) Å^3^
*Z* = 4
Mo *K*α radiation
μ = 1.65 mm^−1^
*T* = 293 K
0.25 × 0.20 × 0.15 mm

#### Data collection

Bruker SMART CCD diffractometer
Absorption correction: multi-scan (*SADABS*; Bruker 2003 ▶) *T*_min_ = 0.68, *T*_max_ = 0.78
15513 measured reflections
5600 independent reflections
2635 reflections with *I* > 2σ(*I*)
*R*_int_ = 0.111

#### Refinement

*R*[*F* ^2^ > 2σ(*F* ^2^)] = 0.065
*wR*(*F* ^2^) = 0.161
*S* = 0.93
5600 reflections
460 parameters
387 restraints
H-atom parameters constrained
Δρ_max_ = 0.59 e Å^−3^
Δρ_min_ = −0.54 e Å^−3^

### 

Data collection: *SMART* (Bruker, 2003 ▶); cell refinement: *SAINT* (Bruker, 2003 ▶); data reduction: *SAINT*; program(s) used to solve structure: *SHELXS97* (Sheldrick, 2008 ▶); program(s) used to refine structure: *SHELXL97* (Sheldrick, 2008 ▶); molecular graphics: *XP* in *SHELXTL* (Sheldrick, 2008 ▶); software used to prepare material for publication: *DIAMOND* (Brandenburg, 2000 ▶).

## Supplementary Material

Crystal structure: contains datablocks I, global. DOI: 10.1107/S1600536810011293/jh2139sup1.cif

Structure factors: contains datablocks I. DOI: 10.1107/S1600536810011293/jh2139Isup2.hkl

Additional supplementary materials:  crystallographic information; 3D view; checkCIF report

## Figures and Tables

**Table 1:** Hydrogen-bond geometry (Å, °)

*D*—H⋯*A*	*D*—H	H⋯*A*	*D*⋯*A*	*D*—H⋯*A*
O13—H13*A*⋯O17^i^	0.85	2.36	2.723 (5)	106
O13—H13*D*⋯O4^ii^	0.85	1.85	2.681 (5)	165
O14—H14*A*⋯O10^iii^	0.85	1.90	2.679 (5)	151
O14—H14*D*⋯O4^iv^	0.85	2.08	2.586 (5)	118
O15—H15*B*⋯O17	0.85	2.06	2.826 (6)	150
O15—H15*C*⋯O10^v^	0.85	1.98	2.607 (5)	130
O16—H16*C*⋯O12	0.85	2.27	2.737 (5)	115
O16—H16*D*⋯O20^i^	0.85	2.37	2.887 (6)	119
O17—H17*B*⋯O6^iii^	0.85	2.52	3.257 (5)	145
O17—H17*C*⋯O11^iii^	0.85	2.34	3.168 (5)	166
O18—H18*B*⋯O8^vi^	0.85	2.31	3.039 (6)	145
O18—H18*D*⋯O19	0.85	2.10	2.710 (6)	128
O19—H19*C*⋯O6^iii^	0.85	2.56	3.087 (5)	121
O19—H19*E*⋯O1^iii^	0.85	2.47	2.867 (5)	109
O20—H20*A*⋯O2^vii^	0.85	2.14	2.938 (6)	156
O20—H20*D*⋯O19	0.85	2.29	2.713 (6)	111

Symmetry codes: (i) 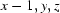; (ii) 
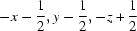
; (iii) 
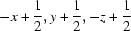
; (iv) 
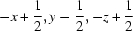
; (v) 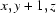; (vi) 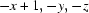; (vii) 
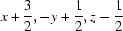
.

**Fig. 1.**
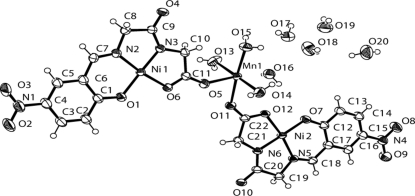
ORTEP plot of complex 1 with atom numbering scheme (Thermal ellipsoids are drawn at 40% probability level).

**Fig. 2.**
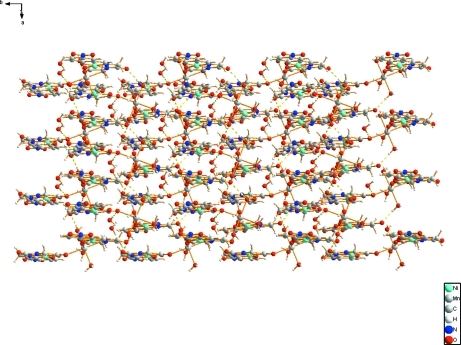
Schematic representation of the hydrogen-bonded (dashed lines)2-D network.

## supplementary crystallographic information

### Comment

Transition metal complexes of salicylaldehyde-peptides and salicylaldehyde-amino acid Schiff base are non-enzymatic models for pyridoxal-amino acid systems, which are of considerable importance as key intermediates in many metabolic reactions of amino acids catalyzed by enzymes (Zabinski *et al.*, 2001; Wetmore *et al.*, 2001; Bkouche-Waksman *et al.*,1988). Considerable effort has been devoted to the preparation, structural characterization, appropriate spectroscopy and magnetic studies of Schiff-base complexes derived from salicylaldehyde and amino acids and reduced salicylidene amino acid (Ganguly *et al.*, 2008), but little attention has been given to Schiff base derived from simple peptides (Zou *et al.*, 2003). Herein, we report the structure study of [Mn(H_2_O)_4_(Ni(C_11_H_8_N_3_O_6_))_2_]_2_^.^4H_2_O (H_2_L= Schiff bases derived from glycylglycine and 5-nitrosalicylaldehyde, C_11_H_10_N_3_O_6_).

The complex crystallizes in the monoclinic system, space group *P*2_1_/*n*. The unit contents consist of one polynuclear molecule Mn(H_2_O)_4_[Ni(L)]_2 _and four water molecules (Fig 1). In each unit, one Mn(H_2_O)_4_^2+^group and two symmetric [Ni(L)]^-^ groups are connected by carboxylate oxygen atoms (O5, O11). The coordination environment of the two Ni(II) centers is approximately square-planar. The deprotonated Schiff base ligand is a triple negatively-charged tetradentate ONNO group, coordinating to the Ni(II) atom *via *one phenolic oxygen atom (Ni1-O1 = 1.860 (3) Å), one deprotonated amide nitrogen atom (Ni1-N2 = 1.832 (4) Å), one imino nitrogen atom (Ni1-N3 =1.816 (4) Å) and one carboxylate oxygen atom (Ni1-O6 =1.879 (3) Å). The values 1.481 (6)Å for the (C8-N2) bond, shorter than the usual C-N single bond and the double bond (C7-N2) length of 1.262 (6)Å agree well with the values of Schiff Base type I. A slight distortion in the square planar geometry of Ni (II) is present (observed bond angles vary from 85.72 (19) and 96.51 (17)° ) The best-fit least-squares plane through the four basal and Ni atoms shows these atoms to be nearly coplanar. The O1-Ni1-N3 angle of 177.46 (18) ° is nearly linear. The ligating atoms at Ni2 are similar to those at Ni1. Two carboxylate oxygen atoms (O5, O11) and four water molecule oxygen atoms (O13, O14, O15, O16) coordinate to the manganese atom forming a distorted octahedron. The Mn—O distances range from 2.125 (4) to 2.252 (3) Å. In the packing scheme of this compound the intermolecular and intramolecular hydrogen bonds plays a very important role. The hydrogen atoms of water bond with the carbonylic, carboxylic, phenolic and nitryl oxygen of the Schiff base ligand (Fig. 2).  The molecules are linked by the hydrogen bond to form a two-dimensional network in the solid state.

### Experimental

The Schiff base was prepared through the condensation of the glycylglycine and 2*-*hydrogen-5-nitrobenzaldehyde. glycylglycine (10 mmol) was dissolved and refluxed in absolute methanol (40 mL) containing LiOH^.^H_2_O (10 mmol). After cooled to room temperature, a solution of 2*-*hydrogen-5-nitrobenzaldehyde (10 mmol) in absolute methanol was added slowly with stirring for 10 min. then NiCl_2_^.^6H_2_O (10 mmol) was added to the HLLi solution and the resulting solution was adjusted to the pH = 9-11 by 1.0 mol/L NaOH solution. After stirring at room temperature for 30 min, the volume was reduced to ca. 5 mL *in vacuo.* Anhydrous ethanol was added to precipitate the product out, which can be recrystallized in methanol solution.(Found: C, 33.4; H, 3.0; N, 10.6. Calc. For C_11_H_12_N_3_O_8_NiNa: C, 33.4; H, 3.1; N, 10.6%.) Na[NiL]^.^2H_2_O (2 mmol) was dissolved in 10 mL water. Then MnCl_2_^.^4H_2_O (1 mmol) was added to the solution with stirring. The resulting crude product was precipitated. It was recrystallized in hot water (90°C) and filtered. The filtrate was allowed to evaporate slowly at room temperature. After several days orange crystals suitable for X-ray diffraction were obtained. (Found: C, 30.4; H, 3.8; N, 9.6. Calc. For C_22_H_32_N_6_O_20_Ni_2_Mn: C, 30.3; H, 3.7; N, 9.6%.)

### Refinement

The water H atoms in the complex were located in a difference Fourier map with a distance restraint of O-H = 0.85 Å and U_iso_(H) =1.5U_eq_(O). All other H atoms were positioned geometrically and constrained as riding atoms, with C-H distances of 0.93–0.97 Å and U_iso_(H) set to 1.2 or 1.5U_eq_(C) of the parent atom.

### Crystal data

**Table:**

[MnNi_2_(C_11_H_8_N_3_O_6_)_2_(H_2_O)_4_]·4H_2_O	*F*(000) = 1788
*M**_r_* = 872.90	*D*_x_ = 1.814 Mg m^−^^3^
Monoclinic, *P*2_1_/*n*	Mo *K*α radiation, λ = 0.71073 Å
Hall symbol: -P 2yn	Cell parameters from 2635 reflections
*a* = 7.250 (1) Å	θ = 1.8–25°
*b* = 11.581 (2) Å	µ = 1.65 mm^−^^1^
*c* = 38.058 (6) Å	*T* = 293 K
β = 90.29 (1)°	Block, orange
*V* = 3195.4 (9) Å^3^	0.25 × 0.20 × 0.15 mm
*Z* = 4	

### Data collection

**Table:**

Bruker SMART CCD diffractometer	5600 independent reflections
Radiation source: fine-focus sealed tube	2635 reflections with *I* > 2σ(*I*)
graphite	*R*_int_ = 0.111
2θ/ω scans	θ_max_ = 25.0°, θ_min_ = 1.8°
Absorption correction: multi-scan (*SADABS*; Bruker 2003)	*h* = −8→8
*T*_min_ = 0.68, *T*_max_ = 0.78	*k* = −13→13
15513 measured reflections	*l* = −36→45

### Refinement

**Table:**

Refinement on *F*^2^	Primary atom site location: structure-invariant direct methods
Least-squares matrix: full	Secondary atom site location: difference Fourier map
*R*[*F*^2^ > 2σ(*F*^2^)] = 0.065	Hydrogen site location: inferred from neighbouring sites
*wR*(*F*^2^) = 0.161	H-atom parameters constrained
*S* = 0.93	*w* = 1/[σ^2^(*F*_o_^2^) + (0.0546*P*)^2^] where *P* = (*F*_o_^2^ + 2*F*_c_^2^)/3
5600 reflections	(Δ/σ)_max_ < 0.001
460 parameters	Δρ_max_ = 0.59 e Å^−^^3^
387 restraints	Δρ_min_ = −0.54 e Å^−^^3^

### Special details

**Table:**

Geometry. All esds (except the esd in the dihedral angle between two l.s. planes) are estimated using the full covariance matrix. The cell esds are taken into account individually in the estimation of esds in distances, angles and torsion angles; correlations between esds in cell parameters are only used when they are defined by crystal symmetry. An approximate (isotropic) treatment of cell esds is used for estimating esds involving l.s. planes.
Refinement. Refinement of *F*^2^ against ALL reflections. The weighted *R*-factor wR and goodness of fit *S* are based on *F*^2^, conventional *R*-factors *R* are based on *F*, with *F* set to zero for negative *F*^2^. The threshold expression of *F*^2^ > σ(*F*^2^) is used only for calculating *R*-factors(gt) etc. and is not relevant to the choice of reflections for refinement. *R*-factors based on *F*^2^ are statistically about twice as large as those based on *F*, and *R*- factors based on ALL data will be even larger.

### Fractional atomic coordinates and isotropic or equivalent isotropic displacement parameters (Å^2^)

**Table:**

	*x*	*y*	*z*	*U*_iso_*/*U*_eq_	
Ni1	−0.13915 (11)	0.32785 (5)	0.362499 (18)	0.0266 (2)	
Ni2	0.13623 (11)	−0.16406 (5)	0.138128 (18)	0.0263 (2)	
Mn1	0.07802 (13)	0.22949 (7)	0.21569 (2)	0.0299 (2)	
C1	−0.2519 (8)	0.2158 (5)	0.42448 (14)	0.0343 (6)	
C2	−0.2786 (8)	0.1139 (5)	0.44456 (14)	0.0363 (7)	
H2A	−0.2503	0.0425	0.4347	0.044*	
C3	−0.3441 (8)	0.1186 (5)	0.47744 (15)	0.0376 (7)	
H3A	−0.3623	0.0506	0.4900	0.045*	
C4	−0.3853 (8)	0.2253 (5)	0.49316 (14)	0.0397 (6)	
C5	−0.3575 (8)	0.3253 (5)	0.47496 (14)	0.0365 (7)	
H5A	−0.3839	0.3956	0.4856	0.044*	
C6	−0.2909 (8)	0.3237 (5)	0.44104 (14)	0.0348 (6)	
C7	−0.2654 (8)	0.4335 (5)	0.42340 (14)	0.0345 (7)	
H7A	−0.2963	0.5003	0.4356	0.041*	
C8	−0.1714 (8)	0.5609 (4)	0.37804 (14)	0.0351 (7)	
H8A	−0.0790	0.6008	0.3920	0.042*	
H8B	−0.2845	0.6056	0.3786	0.042*	
C9	−0.1063 (8)	0.5487 (4)	0.34098 (14)	0.0332 (7)	
C10	−0.0249 (8)	0.4045 (4)	0.29709 (14)	0.0341 (7)	
H10A	0.0979	0.4339	0.2925	0.041*	
H10B	−0.1076	0.4318	0.2788	0.041*	
C11	−0.0235 (8)	0.2740 (5)	0.29821 (14)	0.0363 (6)	
C12	0.2613 (8)	−0.1431 (4)	0.06885 (14)	0.0341 (6)	
C13	0.3061 (8)	−0.0732 (5)	0.03937 (14)	0.0353 (7)	
H13B	0.2925	0.0065	0.0407	0.042*	
C14	0.3657 (8)	−0.1209 (5)	0.00931 (15)	0.0368 (7)	
H14B	0.3987	−0.0733	−0.0093	0.044*	
C15	0.3803 (8)	−0.2388 (5)	0.00598 (14)	0.0378 (6)	
C16	0.3400 (8)	−0.3108 (5)	0.03311 (14)	0.0355 (7)	
H16B	0.3521	−0.3903	0.0304	0.043*	
C17	0.2800 (8)	−0.2648 (5)	0.06529 (14)	0.0334 (6)	
C18	0.2295 (8)	−0.3429 (4)	0.09273 (14)	0.0316 (7)	
H18A	0.2388	−0.4216	0.0882	0.038*	
C19	0.1324 (8)	−0.4005 (4)	0.14912 (13)	0.0322 (7)	
H19A	0.2407	−0.4471	0.1540	0.039*	
H19B	0.0356	−0.4507	0.1403	0.039*	
C20	0.0680 (8)	−0.3401 (4)	0.18241 (14)	0.0313 (7)	
C21	0.0135 (8)	−0.1520 (4)	0.20698 (14)	0.0313 (7)	
H21A	0.0953	−0.1595	0.2271	0.038*	
H21B	−0.1113	−0.1692	0.2144	0.038*	
C22	0.0243 (8)	−0.0315 (4)	0.19229 (14)	0.0326 (6)	
N1	−0.4516 (7)	0.2300 (4)	0.52840 (12)	0.0482 (8)	
N2	−0.2042 (7)	0.4442 (4)	0.39261 (11)	0.0345 (7)	
N3	−0.0881 (6)	0.4422 (3)	0.33140 (11)	0.0326 (7)	
N4	0.4505 (7)	−0.2882 (4)	−0.02607 (12)	0.0437 (8)	
N5	0.1731 (6)	−0.3116 (3)	0.12300 (11)	0.0310 (7)	
N6	0.0683 (6)	−0.2311 (3)	0.17905 (11)	0.0304 (7)	
O1	−0.1939 (5)	0.2064 (3)	0.39269 (9)	0.0344 (7)	
O2	−0.4768 (7)	0.1386 (4)	0.54427 (11)	0.0642 (12)	
O3	−0.4846 (7)	0.3229 (4)	0.54206 (11)	0.0600 (11)	
O4	−0.0787 (6)	0.6368 (3)	0.32280 (10)	0.0368 (10)	
O5	0.0188 (6)	0.2181 (3)	0.27154 (10)	0.0417 (7)	
O6	−0.0703 (5)	0.2253 (3)	0.32661 (9)	0.0366 (7)	
O7	0.2050 (5)	−0.0921 (3)	0.09717 (9)	0.0348 (7)	
O8	0.4990 (6)	−0.2206 (3)	−0.04983 (10)	0.0560 (11)	
O9	0.4621 (6)	−0.3915 (3)	−0.02960 (11)	0.0560 (11)	
O10	0.0257 (5)	−0.3997 (3)	0.20923 (9)	0.0341 (10)	
O11	−0.0247 (5)	0.0516 (3)	0.21069 (9)	0.0353 (7)	
O12	0.0853 (5)	−0.0228 (3)	0.16104 (9)	0.0316 (7)	
O13	−0.1904 (5)	0.2964 (3)	0.20329 (11)	0.0541 (10)	
H13A	−0.2458	0.3118	0.2223	0.081*	
H13D	−0.2508	0.2456	0.1919	0.081*	
O14	0.3433 (5)	0.1613 (3)	0.22748 (10)	0.0437 (9)	
H14A	0.3580	0.1596	0.2496	0.066*	
H14D	0.4251	0.2044	0.2184	0.066*	
O15	0.1896 (6)	0.4015 (3)	0.21661 (10)	0.0427 (10)	
H15B	0.3018	0.3994	0.2102	0.064*	
H15C	0.1284	0.4438	0.2025	0.064*	
O16	0.1345 (6)	0.2113 (3)	0.15781 (9)	0.0425 (10)	
H16C	0.2093	0.1558	0.1547	0.064*	
H16D	0.0341	0.1973	0.1470	0.064*	
O17	0.5613 (6)	0.4713 (3)	0.21007 (11)	0.0573 (15)	
H17B	0.5287	0.5210	0.1948	0.086*	
H17C	0.5642	0.5026	0.2302	0.086*	
O18	0.3366 (6)	0.3427 (4)	0.11301 (11)	0.0700 (17)	
H18B	0.3322	0.3135	0.0925	0.105*	
H18D	0.4479	0.3445	0.1201	0.105*	
O19	0.6018 (6)	0.4872 (3)	0.13746 (11)	0.0641 (16)	
H19C	0.6778	0.5328	0.1473	0.096*	
H19E	0.5347	0.5248	0.1231	0.096*	
O20	0.8821 (7)	0.3487 (4)	0.11641 (12)	0.0738 (17)	
H20D	0.7738	0.3294	0.1225	0.089*	
H20A	0.8938	0.3398	0.0944	0.111*	

### Atomic displacement parameters (Å^2^)

**Table:**

	*U*^11^	*U*^22^	*U*^33^	*U*^12^	*U*^13^	*U*^23^
Ni1	0.0393 (5)	0.0211 (3)	0.0196 (4)	0.0026 (4)	0.0049 (3)	0.0005 (3)
Ni2	0.0400 (5)	0.0171 (3)	0.0220 (4)	0.0029 (4)	0.0017 (3)	0.0001 (3)
Mn1	0.0426 (6)	0.0215 (4)	0.0256 (5)	0.0016 (4)	0.0047 (4)	0.0000 (4)
C1	0.0433 (13)	0.0324 (10)	0.0274 (10)	−0.0038 (12)	0.0070 (11)	0.0030 (9)
C2	0.0443 (15)	0.0348 (11)	0.0298 (12)	−0.0036 (14)	0.0056 (13)	0.0047 (11)
C3	0.0459 (16)	0.0371 (11)	0.0298 (12)	−0.0078 (14)	0.0054 (13)	0.0061 (11)
C4	0.0496 (13)	0.0406 (11)	0.0290 (10)	−0.0097 (12)	0.0070 (11)	0.0015 (9)
C5	0.0453 (15)	0.0364 (12)	0.0280 (11)	−0.0070 (14)	0.0056 (13)	−0.0010 (10)
C6	0.0435 (13)	0.0340 (10)	0.0270 (10)	−0.0038 (12)	0.0064 (11)	−0.0004 (9)
C7	0.0456 (15)	0.0318 (11)	0.0262 (11)	0.0000 (14)	0.0100 (13)	−0.0031 (10)
C8	0.0508 (15)	0.0250 (11)	0.0295 (11)	0.0019 (14)	0.0100 (13)	−0.0024 (10)
C9	0.0478 (15)	0.0230 (10)	0.0289 (12)	−0.0008 (13)	0.0093 (13)	0.0001 (9)
C10	0.0487 (15)	0.0253 (10)	0.0283 (12)	−0.0046 (14)	0.0134 (13)	−0.0020 (10)
C11	0.0538 (13)	0.0254 (10)	0.0299 (9)	−0.0030 (12)	0.0157 (11)	−0.0046 (10)
C12	0.0466 (13)	0.0292 (10)	0.0265 (10)	−0.0019 (12)	0.0043 (11)	0.0014 (9)
C13	0.0474 (15)	0.0316 (12)	0.0270 (12)	−0.0032 (14)	0.0034 (13)	0.0023 (10)
C14	0.0479 (16)	0.0354 (11)	0.0272 (12)	−0.0055 (14)	0.0026 (13)	0.0010 (11)
C15	0.0496 (13)	0.0355 (10)	0.0284 (10)	−0.0063 (12)	0.0025 (11)	−0.0018 (9)
C16	0.0456 (15)	0.0319 (12)	0.0289 (11)	−0.0040 (14)	0.0031 (12)	−0.0031 (10)
C17	0.0440 (13)	0.0289 (10)	0.0273 (10)	−0.0019 (12)	0.0025 (11)	−0.0010 (9)
C18	0.0443 (15)	0.0235 (12)	0.0270 (11)	−0.0010 (14)	0.0024 (12)	−0.0038 (10)
C19	0.0475 (15)	0.0201 (11)	0.0289 (11)	0.0005 (14)	0.0033 (13)	−0.0006 (10)
C20	0.0469 (15)	0.0200 (10)	0.0271 (11)	0.0006 (13)	0.0028 (13)	0.0004 (10)
C21	0.0449 (15)	0.0207 (10)	0.0284 (12)	−0.0008 (13)	0.0071 (13)	−0.0008 (10)
C22	0.0470 (13)	0.0206 (9)	0.0302 (11)	−0.0008 (12)	0.0068 (11)	−0.0020 (9)
N1	0.0628 (17)	0.0497 (13)	0.0323 (13)	−0.0140 (15)	0.0127 (14)	0.0000 (11)
N2	0.0492 (16)	0.0281 (10)	0.0263 (11)	0.0012 (13)	0.0098 (12)	−0.0012 (9)
N3	0.0476 (15)	0.0235 (9)	0.0269 (11)	−0.0038 (13)	0.0113 (13)	−0.0022 (9)
N4	0.0584 (17)	0.0394 (12)	0.0333 (13)	−0.0085 (15)	0.0088 (14)	−0.0036 (11)
N5	0.0449 (15)	0.0201 (10)	0.0279 (10)	−0.0002 (13)	0.0050 (12)	−0.0022 (9)
N6	0.0456 (15)	0.0191 (9)	0.0267 (11)	−0.0012 (13)	0.0060 (12)	0.0002 (9)
O1	0.0462 (18)	0.0298 (12)	0.0272 (11)	−0.0009 (15)	0.0069 (13)	0.0017 (10)
O2	0.096 (3)	0.0608 (15)	0.0361 (19)	−0.018 (2)	0.025 (2)	0.0044 (16)
O3	0.080 (3)	0.0618 (15)	0.038 (2)	−0.005 (2)	0.027 (2)	−0.0038 (16)
O4	0.052 (2)	0.0251 (14)	0.0339 (18)	−0.0018 (18)	0.0044 (18)	0.0058 (14)
O5	0.0627 (16)	0.0301 (13)	0.0324 (10)	−0.0030 (15)	0.0174 (11)	−0.0070 (10)
O6	0.0546 (18)	0.0226 (11)	0.0327 (11)	−0.0025 (15)	0.0165 (14)	−0.0039 (10)
O7	0.0515 (18)	0.0264 (12)	0.0267 (11)	−0.0008 (15)	0.0055 (13)	0.0021 (10)
O8	0.082 (3)	0.0493 (17)	0.0368 (17)	−0.012 (2)	0.0166 (19)	−0.0006 (16)
O9	0.083 (3)	0.0416 (14)	0.043 (2)	−0.008 (2)	0.016 (2)	−0.0106 (15)
O10	0.048 (2)	0.0242 (16)	0.0300 (16)	0.0045 (18)	0.0046 (17)	0.0047 (13)
O11	0.0509 (15)	0.0233 (9)	0.0319 (14)	−0.0016 (11)	0.0061 (13)	−0.0050 (10)
O12	0.0471 (18)	0.0172 (11)	0.0305 (12)	−0.0018 (14)	0.0082 (13)	−0.0013 (10)
O13	0.0444 (14)	0.0422 (19)	0.076 (2)	0.0112 (14)	−0.0082 (16)	−0.014 (2)
O14	0.0414 (14)	0.0468 (19)	0.043 (2)	0.0072 (14)	0.0042 (14)	0.003 (2)
O15	0.055 (2)	0.0285 (13)	0.045 (2)	−0.0078 (14)	−0.001 (2)	0.0030 (14)
O16	0.058 (2)	0.040 (2)	0.0293 (12)	−0.0019 (19)	0.0033 (14)	−0.0009 (14)
O17	0.070 (3)	0.037 (2)	0.066 (3)	0.001 (2)	0.015 (3)	0.001 (2)
O18	0.082 (4)	0.080 (3)	0.047 (3)	−0.024 (3)	0.010 (3)	−0.013 (3)
O19	0.087 (4)	0.044 (3)	0.062 (3)	−0.004 (3)	0.007 (3)	0.001 (2)
O20	0.077 (4)	0.093 (4)	0.051 (3)	0.007 (3)	0.024 (3)	0.007 (3)

### Geometric parameters (Å, °)

**Table:**

Ni1—N3	1.816 (4)	C13—C14	1.344 (7)
Ni1—N2	1.832 (4)	C13—H13B	0.9300
Ni1—O1	1.860 (3)	C14—C15	1.376 (7)
Ni1—O6	1.879 (3)	C14—H14B	0.9299
Ni2—N6	1.811 (4)	C15—C16	1.361 (7)
Ni2—N5	1.823 (4)	C15—N4	1.442 (7)
Ni2—O7	1.839 (4)	C16—C17	1.407 (7)
Ni2—O12	1.891 (3)	C16—H16B	0.9300
Mn1—O14	2.125 (4)	C17—C18	1.431 (7)
Mn1—O13	2.145 (4)	C18—N5	1.277 (6)
Mn1—O15	2.150 (3)	C18—H18A	0.9299
Mn1—O5	2.174 (4)	C19—N5	1.462 (6)
Mn1—O11	2.198 (3)	C19—C20	1.522 (7)
Mn1—O16	2.252 (3)	C19—H19A	0.9701
C1—O1	1.287 (6)	C19—H19B	0.9698
C1—C2	1.421 (7)	C20—N6	1.268 (6)
C1—C6	1.428 (7)	C20—O10	1.271 (6)
C2—C3	1.341 (7)	C21—N6	1.460 (6)
C2—H2A	0.9300	C21—C22	1.506 (7)
C3—C4	1.405 (8)	C21—H21A	0.9701
C3—H3A	0.9300	C21—H21B	0.9701
C4—C5	1.365 (7)	C22—O11	1.243 (6)
C4—N1	1.428 (7)	C22—O12	1.275 (6)
C5—C6	1.381 (7)	N1—O3	1.219 (6)
C5—H5A	0.9300	N1—O2	1.233 (6)
C6—C7	1.449 (7)	N4—O9	1.207 (6)
C7—N2	1.262 (6)	N4—O8	1.248 (5)
C7—H7A	0.9300	O13—H13A	0.8499
C8—N2	1.481 (6)	O13—H13D	0.8501
C8—C9	1.496 (7)	O14—H14A	0.8501
C8—H8A	0.9700	O14—H14D	0.8499
C8—H8B	0.9700	O15—H15B	0.8501
C9—O4	1.249 (6)	O15—H15C	0.8502
C9—N3	1.294 (6)	O16—H16C	0.8497
C10—N3	1.453 (6)	O16—H16D	0.8496
C10—C11	1.512 (7)	O17—H17B	0.8503
C10—H10A	0.9700	O17—H17C	0.8491
C10—H10B	0.9700	O18—H18B	0.8499
C11—O5	1.243 (6)	O18—H18D	0.8500
C11—O6	1.267 (6)	O19—H19C	0.8500
C12—O7	1.296 (6)	O19—H19E	0.8500
C12—C13	1.422 (7)	O20—H20D	0.8499
C12—C17	1.422 (7)	O20—H20A	0.8501
			
N3—Ni1—N2	85.72 (19)	C13—C14—H14B	119.4
N3—Ni1—O1	177.46 (18)	C15—C14—H14B	119.9
N2—Ni1—O1	96.51 (17)	C16—C15—C14	121.5 (5)
N3—Ni1—O6	86.06 (17)	C16—C15—N4	118.5 (5)
N2—Ni1—O6	171.67 (17)	C14—C15—N4	119.9 (5)
O1—Ni1—O6	91.69 (15)	C15—C16—C17	119.8 (5)
N6—Ni2—N5	84.86 (19)	C15—C16—H16B	120.1
N6—Ni2—O7	178.46 (17)	C17—C16—H16B	120.1
N5—Ni2—O7	96.66 (17)	C16—C17—C12	119.3 (5)
N6—Ni2—O12	85.41 (17)	C16—C17—C18	118.5 (5)
N5—Ni2—O12	170.27 (17)	C12—C17—C18	122.2 (5)
O7—Ni2—O12	93.07 (15)	N5—C18—C17	124.3 (5)
O14—Mn1—O13	179.20 (16)	N5—C18—H18A	117.9
O14—Mn1—O15	90.04 (15)	C17—C18—H18A	117.8
O13—Mn1—O15	90.57 (15)	N5—C19—C20	107.8 (4)
O14—Mn1—O5	87.37 (15)	N5—C19—H19A	110.9
O13—Mn1—O5	93.08 (16)	C20—C19—H19A	110.3
O15—Mn1—O5	96.69 (14)	N5—C19—H19B	109.6
O14—Mn1—O11	88.61 (14)	C20—C19—H19B	109.8
O13—Mn1—O11	90.74 (15)	H19A—C19—H19B	108.4
O15—Mn1—O11	175.37 (14)	N6—C20—O10	128.4 (5)
O5—Mn1—O11	87.67 (14)	N6—C20—C19	111.9 (5)
O14—Mn1—O16	90.16 (15)	O10—C20—C19	119.7 (4)
O13—Mn1—O16	89.30 (16)	N6—C21—C22	107.3 (4)
O15—Mn1—O16	91.85 (14)	N6—C21—H21A	110.6
O5—Mn1—O16	171.11 (13)	C22—C21—H21A	110.1
O11—Mn1—O16	83.72 (13)	N6—C21—H21B	110.0
O1—C1—C2	118.8 (5)	C22—C21—H21B	110.4
O1—C1—C6	123.7 (5)	H21A—C21—H21B	108.5
C2—C1—C6	117.5 (5)	O11—C22—O12	124.5 (5)
C3—C2—C1	121.2 (5)	O11—C22—C21	119.5 (5)
C3—C2—H2A	119.4	O12—C22—C21	116.0 (4)
C1—C2—H2A	119.4	O3—N1—O2	121.2 (5)
C2—C3—C4	120.7 (5)	O3—N1—C4	120.2 (5)
C2—C3—H3A	119.7	O2—N1—C4	118.6 (5)
C4—C3—H3A	119.7	C7—N2—C8	119.7 (4)
C5—C4—C3	119.8 (5)	C7—N2—Ni1	127.0 (4)
C5—C4—N1	119.7 (5)	C8—N2—Ni1	113.3 (3)
C3—C4—N1	120.5 (5)	C9—N3—C10	125.0 (4)
C4—C5—C6	121.1 (5)	C9—N3—Ni1	119.4 (4)
C4—C5—H5A	119.5	C10—N3—Ni1	115.6 (3)
C6—C5—H5A	119.5	O9—N4—O8	121.4 (5)
C5—C6—C1	119.7 (5)	O9—N4—C15	120.8 (5)
C5—C6—C7	117.8 (5)	O8—N4—C15	117.8 (4)
C1—C6—C7	122.5 (5)	C18—N5—C19	118.7 (4)
N2—C7—C6	124.2 (5)	C18—N5—Ni2	126.8 (4)
N2—C7—H7A	117.9	C19—N5—Ni2	114.5 (3)
C6—C7—H7A	117.9	C20—N6—C21	123.4 (4)
N2—C8—C9	108.6 (4)	C20—N6—Ni2	120.9 (4)
N2—C8—H8A	110.0	C21—N6—Ni2	115.7 (3)
C9—C8—H8A	110.0	C1—O1—Ni1	126.0 (3)
N2—C8—H8B	110.0	C11—O5—Mn1	144.8 (4)
C9—C8—H8B	110.0	C11—O6—Ni1	114.4 (3)
H8A—C8—H8B	108.4	C12—O7—Ni2	125.9 (3)
O4—C9—N3	127.3 (5)	C22—O11—Mn1	132.6 (4)
O4—C9—C8	119.8 (5)	C22—O12—Ni2	115.6 (3)
N3—C9—C8	112.9 (5)	Mn1—O13—H13A	108.7
N3—C10—C11	106.1 (4)	Mn1—O13—H13D	109.1
N3—C10—H10A	110.5	H13A—O13—H13D	109.5
C11—C10—H10A	110.5	Mn1—O14—H14A	109.1
N3—C10—H10B	110.5	Mn1—O14—H14D	109.2
C11—C10—H10B	110.5	H14A—O14—H14D	109.5
H10A—C10—H10B	108.7	Mn1—O15—H15B	109.2
O5—C11—O6	122.2 (5)	Mn1—O15—H15C	109.2
O5—C11—C10	119.9 (5)	H15B—O15—H15C	109.5
O6—C11—C10	117.8 (5)	Mn1—O16—H16C	109.1
O7—C12—C13	118.1 (5)	Mn1—O16—H16D	109.4
O7—C12—C17	124.1 (5)	H16C—O16—H16D	109.5
C13—C12—C17	117.8 (5)	H17B—O17—H17C	109.5
C14—C13—C12	120.9 (5)	H18B—O18—H18D	109.5
C14—C13—H13B	119.2	H19C—O19—H19E	109.5
C12—C13—H13B	119.8	H20D—O20—H20A	109.5
C13—C14—C15	120.8 (5)		

### Hydrogen-bond geometry (Å, °)

**Table:**

*D*—H···*A*	*D*—H	H···*A*	*D*···*A*	*D*—H···*A*
O13—H13A···O17^i^	0.85	2.36	2.723 (5)	106
O13—H13D···O4^ii^	0.85	1.85	2.681 (5)	165
O14—H14A···O10^iii^	0.85	1.90	2.679 (5)	151
O14—H14D···O4^iv^	0.85	2.08	2.586 (5)	118
O15—H15B···O17	0.85	2.06	2.826 (6)	150
O15—H15C···O10^v^	0.85	1.98	2.607 (5)	130
O16—H16C···O12	0.85	2.27	2.737 (5)	115
O16—H16D···O20^i^	0.85	2.37	2.887 (6)	119
O17—H17B···O6^iii^	0.85	2.52	3.257 (5)	145
O17—H17C···O11^iii^	0.85	2.34	3.168 (5)	166
O18—H18B···O8^vi^	0.85	2.31	3.039 (6)	145
O18—H18D···O19	0.85	2.10	2.710 (6)	128
O19—H19C···O6^iii^	0.85	2.56	3.087 (5)	121
O19—H19E···O1^iii^	0.85	2.47	2.867 (5)	109
O20—H20A···O2^vii^	0.85	2.14	2.938 (6)	156
O20—H20D···O19	0.85	2.29	2.713 (6)	111

Symmetry codes: (i) *x*−1, *y*, *z*; (ii) −*x*−1/2, *y*−1/2, −*z*+1/2; (iii) −*x*+1/2, *y*+1/2, −*z*+1/2; (iv) −*x*+1/2, *y*−1/2, −*z*+1/2; (v) *x*, *y*+1, *z*; (vi) −*x*+1, −*y*, −*z*; (vii) *x*+3/2, −*y*+1/2, *z*−1/2.
